# Blood Rheology: Key Parameters, Impact on Blood Flow, Role in Sickle Cell Disease and Effects of Exercise

**DOI:** 10.3389/fphys.2019.01329

**Published:** 2019-10-17

**Authors:** Elie Nader, Sarah Skinner, Marc Romana, Romain Fort, Nathalie Lemonne, Nicolas Guillot, Alexandra Gauthier, Sophie Antoine-Jonville, Céline Renoux, Marie-Dominique Hardy-Dessources, Emeric Stauffer, Philippe Joly, Yves Bertrand, Philippe Connes

**Affiliations:** ^1^Laboratory LIBM EA7424, Team “Vascular Biology and Red Blood Cell”, University of Lyon 1, Lyon, France; ^2^Laboratory of Excellence GR-Ex, Paris, France; ^3^Biologie Intégrée du Globule Rouge, Université de Paris, UMR_S1134, BIGR, INSERM, F-75015, Paris, France; ^4^Biologie Intégrée du Globule Rouge, The Université des Antilles, UMR_S1134, BIGR, F- 97157, Pointe-a-Pitre, France; ^5^Département de Médecine, Hôpital Edouard Herriot, Hospices Civils de Lyon, Lyon, France; ^6^Unité Transversale de la Drépanocytose, Hôpital de Pointe-a-Pitre, Hôpital Ricou, Pointe-a-Pitre, France; ^7^Laboratoire Carmen INSERM 1060, INSA Lyon, Université Claude Bernard Lyon 1, Université de Lyon, Villeurbanne, France; ^8^d’Hématologie et d’Oncologie Pédiatrique, Hospices Civils de Lyon, Lyon, France; ^9^Laboratoire ACTES EA 3596, The Université des Antilles, Pointe-a-Pitre, France; ^10^Laboratoire de Biochimie et de Biologie Moleìculaire, UF de Biochimie des Pathologies Eìrythrocytaires, Centre de Biologie et de Pathologie Est, Hospices Civils de Lyon, Lyon, France; ^11^Centre de Médecine du Sommeil et des Maladies Respiratoires, Hospices Civils de Lyon, Hôpital de la Croix Rousse, Lyon, France

**Keywords:** blood rheology, red blood cell deformability, red blood cell aggregation, sickle cell disease, exercise

## Abstract

Blood viscosity is an important determinant of local flow characteristics, which exhibits shear thinning behavior: it decreases exponentially with increasing shear rates. Both hematocrit and plasma viscosity influence blood viscosity. The shear thinning property of blood is mainly attributed to red blood cell (RBC) rheological properties. RBC aggregation occurs at low shear rates, and increases blood viscosity and depends on both cellular (RBC aggregability) and plasma factors. Blood flow in the microcirculation is highly dependent on the ability of RBC to deform, but RBC deformability also affects blood flow in the macrocirculation since a loss of deformability causes a rise in blood viscosity. Indeed, any changes in one or several of these parameters may affect blood viscosity differently. Poiseuille’s Law predicts that any increase in blood viscosity should cause a rise in vascular resistance. However, blood viscosity, through its effects on wall shear stress, is a key modulator of nitric oxide (NO) production by the endothelial NO-synthase. Indeed, any increase in blood viscosity should promote vasodilation. This is the case in healthy individuals when vascular function is intact and able to adapt to blood rheological strains. However, in sickle cell disease (SCD) vascular function is impaired. In this context, any increase in blood viscosity can promote vaso-occlusive like events. We previously showed that sickle cell patients with high blood viscosity usually have more frequent vaso-occlusive crises than those with low blood viscosity. However, while the deformability of RBC decreases during acute vaso-occlusive events in SCD, patients with the highest RBC deformability at steady-state have a higher risk of developing frequent painful vaso-occlusive crises. This paradox seems to be due to the fact that in SCD RBC with the highest deformability are also the most adherent, which would trigger vaso-occlusion. While acute, intense exercise may increase blood viscosity in healthy individuals, recent works conducted in sickle cell patients have shown that light cycling exercise did not cause dramatic changes in blood rheology. Moreover, regular physical exercise has been shown to decrease blood viscosity in sickle cell mice, which could be beneficial for adequate blood flow and tissue perfusion.

## Blood Flow Resistance and the Cardiovascular System

Flow velocity in a given tube depends on pressure and flow resistance. According to Poiseuille’s Law (Poiseuille, 1835), flow resistance depends on the geometry of the tube [length (L) and radius of the tube (r)] and the fluid’s viscosity (η), and is calculated using the following formula:

$$R=\frac{8\,\eta \,L}{\pi \,r^{4}}$$

When applying Poiseuille’s Law to the cardiovascular system, one must consider the radius and the length of the vessels, and the viscosity of the blood. The dimensions of the vascular system (most notably the radius, which is raised to the fourth power) play a more important role in determining vascular resistance than blood viscosity does. However, several works conducted in the past 10–15 years have shown that, in a physiological context, the parameters of this equation cannot be considered to be truly independent of each other. This is because vessels are not rigid tubes; they can change their diameters in response to various physiological stimuli. One of the most important molecules that promotes an augmentation in vascular diameter (i.e., vasodilation) is nitric oxide (NO). Martini et al. (2005), Tsai et al. (2005), Intaglietta (2009), and Sriram et al. (2012) showed that mild to moderate increases in hematocrit and blood viscosity did not result in a rise in vascular resistance or blood pressure, but actually caused the opposite effect. They also showed that increasing blood viscosity promoted the activation of endothelial NO-synthase through shear stress-dependent mechanisms, resulting in higher NO production, compensatory vasodilation, and decreased arterial pressure. However, evidence shows that these vascular adaptations can only occur in a functioning vascular system with a healthy endothelium. When vascular dysfunction is present, vasodilation is impaired. Therefore, a rise in blood viscosity is not accompanied by an increase in vasodilation. As a result, vascular resistance and arterial pressure increase (Vazquez et al., 2010; Salazar Vazquez et al., 2011). Although the role of blood viscosity in vascular adaptations is often ignored, these studies clearly demonstrate that vascular geometry and blood viscosity should not be considered separately when studying the regulation of vascular resistance in healthy populations or in people with cardiovascular diseases.

## Blood is Not a Simple Fluid

Whole blood is a two-phase liquid, composed of cellular elements suspended in plasma, an aqueous solution containing organic molecules, proteins, and salts (Baskurt and Meiselman, 2003). The cellular phase of blood includes, erythrocytes, leukocytes, and platelets. White blood cells and platelets can affect blood rheology, but under normal conditions, red blood cells (RBCs) have the biggest influence (Pop et al., 2002). Blood rheological properties are determined by the physical properties of these two phases and their relative contribution to total blood volume.

Blood is a non-Newtonian, shear thinning fluid with thixotropic and viscoelastic properties. Many cardiovascular handbooks consider blood viscosity values between 3.5 and 5.5 cP to be normal. However, blood viscosity cannot be summarized by a single value. Due to the shear thinning property of blood, which is dependent on RBC rheological properties, the viscosity of this fluid changes depending on the hemodynamic conditions. The same blood can have a viscosity value of 60 cP at a shear rate of 0.1 s^–1^, whereas the viscosity would be 5 or 6 cP at a shear rate of 200 s^–1^. This means that blood viscosity is different in the large arteries, the veins, and the microcirculation, where shear rate can vary from few s^–1^ to more than 1000 s^–1^ (Connes et al., 2016). Blood viscosity depends on several factors: hematocrit, plasma viscosity, the ability of RBCs to deform under flow, and RBC aggregation-disaggregation properties (Baskurt and Meiselman, 2003; Cokelet and Meiselman, 2007).

### Effect of Hematocrit

Whole blood viscosity is dependent on the number (and volume) of erythrocytes in the blood, and is thus linearly related to hematocrit (Chien et al., 1975). The impact of hematocrit on blood viscosity is much higher at low shear rates (veins for instance) than at high shear rate (arteries for instance) (Cokelet and Meiselman, 2007). At high shear rate, it is estimated that a rise of hematocrit of one unit would cause an increase of blood viscosity of 4% (if RBC rheological properties remain the same).

### Plasma Viscosity

Plasma is a newtonian fluid, which means that its viscosity does not vary with shear rate. The viscosity of plasma is dependent on the concentration of plasma proteins, such as fibrinogen, α1-globulins, α2-globulins, β-globulins, and γ-globulins (Connes et al., 2008). Any elevation in the concentration of these proteins can cause plasma, and thus whole blood, viscosity to increase (Kesmarky et al., 2008). Normal plasma at 37 degrees Celsius has a viscosity of around 1.2–1.3 cP, but these values may be higher in various inflammatory, metabolic, or cardiovascular diseases (Kesmarky et al., 2008). Furthermore, increased plasma viscosity is associated with higher rates of adverse clinical events in unstable angina pectoris and stroke (Kesmarky et al., 2008).

### RBC Deformability

Red blood cell deformability is another important determinant of blood viscosity. RBC deformability depends on several factors, including internal (cytosolic) viscosity (mainly determined by the mean cell hemoglobin concentration), membrane viscoelasticity (which is dependent on cytoskeleton proteins and lipid bilayer properties), and the surface-area-to-volume ratio (also called cell sphericity) (Clark et al., 1983; Renoux et al., 2019). At low shear rates, rigid RBCs are less likely to aggregate than deformable RBCs. Therefore, a loss of RBC deformability at very low shear rates (less than 1 s^–1^) results in a decrease in blood viscosity (Chien et al., 1970). In contrast, at shear rates above 1 s^–1^, a decrease in RBC deformability causes blood viscosity to increase (Chien et al., 1970).

Initial experiments done to analyze RBC deformability in blood flow were conducted in the 1970s and 1980s using microtubes (Goldsmith et al., 1972) and rheoscopes (Fischer et al., 1978). These studies demonstrated that, as shear stress increases, normal RBCs align with the direction of flow by deforming into an elliptical shape via a “tank tread-like” motion of the cell membrane around the cytoplasm (Schmid-Schonbein et al., 1969; Goldsmith et al., 1972; Fischer et al., 1978). Rigid RBCs, on the other hand, cannot properly deform into an ellipse and remain perpendicular to blood flow, consequently increasing vascular resistance. In these fundamental experiments, RBCs were suspended in solutions, such as dextrose, with higher viscosities than the internal viscosity of a RBC (Goldsmith et al., 1972; Fischer et al., 1978). However, when a RBC is flowing in plasma *in vivo*, the plasma viscosity is lower than the viscosity of the erythrocyte’s cytosol. This is important because recent experiments reveal that the “tank-treading” behavior of erythrocytes does not occur when RBCs are suspended in solutions with lower viscosities that are more similar to plasma viscosity *in vivo* (Dupire et al., 2012). Instead, observations by Lanotte et al. (2016) showed that RBCs display a wide variety of cell shapes for any given flow condition. For example, erythrocytes in dilute suspensions behave like rigid oblate ellipsoids at low shear rates (<1 s^–1^). Then, as shear rates increase, the erythrocytes successively tumble, roll, and deform into stomatocytes, and eventually adopt highly deformed poly-lobed. The findings of Lanotte et al. (2016) suggest that the pathological alterations of multiple parameters, including plasma composition, erythrocyte cytosol viscosity, and/or membrane mechanical properties could contribute to pathological blood rheology and flow. Further research should be conducted to determine how decreased RBC deformability could affect RBC shape transitions.

Red blood cell deformability is also a key determinant of blood flow in the microcirculation. RBCs are bi-concave disks with an average diameter of around 7–8 μm. Capillaries can have a diameter of less than 5 μm. Therefore, RBC must be highly deformable to pass through the narrowest vessels of the microcirculation. A 15% decrease in RBC deformability has been shown to cause a 75% increase in whole flow resistance in isolated perfused rat hind limbs (Baskurt et al., 2004). Moreover, when dog lungs were perfused with rigid RBC, whole pulmonary arterial pressure increased, and the main increase was localized in the microcirculation (Hakim, 1988; Raj et al., 1991). Indeed, any decrease of RBC deformability may affect flow resistance, tissue perfusion, and oxygenation (Parthasarathi and Lipowsky, 1999).

### RBC Aggregation

Red blood cell aggregation is the reversible formation of three-dimensional stacks of RBC, called “rouleaux,” which takes place at low shear rates. This unique process requires low energy and is reversible under high shear rate conditions. RBC aggregation depends on both plasma and cellular factors. Initially, most research on RBC aggregation formation was focused on the effects of protein levels, polymer type, and concentration. More recent research has shown that RBC cellular properties can also modulate a cell’s intrinsic tendency to aggregate (termed RBC aggregability) (Baskurt and Meiselman, 2009). For example, RBC surface properties, including surface charge and glycocalyx depth, also play an important role in this process. Two models have been proposed to explain RBC aggregation mechanisms (Meiselman et al., 2007), the depletion model and the bridging model. The depletion model suggests that RBC aggregates are formed due to osmotic pressure from surrounding plasma proteins or other macromolecules. The bridging model suggests that aggregates form due to “crossbridges,” made of plasma proteins or other macromolecules (Rampling et al., 2004). Fibrinogen is the most physiologically relevant macromolecule that promotes RBC aggregation (Rampling et al., 2004), but other molecules, including thrombospondin and the von-Willebrand factor, also play a role (Nader et al., 2017).

The impact of RBC aggregation on blood flow, tissue perfusion and vascular resistance is complex and depends on the vascular areas where RBC aggregates are flowing. RBC aggregates usually form in low shear rate areas, such as in veins or in bifurcations. Therefore, increased RBC aggregation would cause a dramatic increase of blood viscosity in these zones. RBC aggregates disaggregate in high shear rate areas, such as in arteries and arterioles. However, it has been demonstrated that some RBC aggregates can persist in large arteries and affect flow dynamics. Increased RBC aggregation has been shown to promote RBC axial migration in these vessels, which in turn increases the cell free layer width (Baskurt and Meiselman, 2007). This latter phenomenon has three main consequences: (i) a decrease of apparent dynamic blood viscosity and of flow resistance, (ii) a decrease of wall shear stress, which in turn results in a lower activation of endothelial NO-synthase, lower NO production, and less vasodilation, and (iii) an increase of plasma skimming phenomena at bifurcations, which in turn lowers microcirculatory hematocrit and blood viscosity (Fharaeus and Fahraeaus–Lindqvist effects).

At the microcirculatory level, persisting RBC aggregates may increase pre-capillary resistance. Experiments performed on large glass tubes also shown that the consequences of RBC aggregation on flow resistance are dependent on the orientation of the tube (vertical vs. horizontal) (Cokelet and Goldsmith, 1991). In horizontal tubes, elevated RBC aggregation increases RBC sedimentation, thereby increasing flow resistance. On the other hand, in vertical tubes RBC aggregation increases RBC axial migration and facilitates blood flow. For this reason, the consequences of increased RBC aggregation on blood flow are difficult to predict. However, experiments done on whole organs, such as guinea pig hearts, reported that gradually increasing RBC aggregation causes a 3-phase evolution of blood flow resistance (Yalcin et al., 2005). First, when RBC aggregation was increased by 50–100%, the authors observed a rise in blood flow resistance. Next, a 100–150% increase in RBC aggregation caused blood flow resistance to decrease. Finally, an increase in RBC aggregation of over 150% caused blood flow resistance to increase once again. Overall, predicting the consequences of increased RBC aggregation *in vivo* appears to be complex. However, clinical works performed to study aggregation in cardiovascular, metabolic, or inflammatory diseases consistently show that people with these diseases generally have higher RBC aggregation levels than healthy individuals, and the elevated aggregation contributes to the development of adverse disease outcomes (Totsimon et al., 2017; Biro et al., 2018; Brun et al., 2018; Ko et al., 2018; Lapoumeroulie et al., 2019; Piecuch et al., 2019; Sheremet’ev et al., 2019).

## Sickle Cell Disease, Blood Rheology and Vascular Dysfunction

Sickle cell disease (SCD) is the most prevalent genetic disease in the world. Sickle cell anemia (SCA) is by far the most common form of SCD, followed by hemoglobin SC disease (SC) (Piel et al., 2010). It is estimated that more than 300,000 children are born each year with a severe inherited hemoglobinopathy, over 80% of these infants are born in low-or middle-income countries, and approximately 220,000 are affected by SCA (Piel et al., 2010).

### Sickle Cell Anemia as a Hemorheological Disease

Sickle cell anemia is caused by a single nucleotide mutation (adenine → thymine) in exon I of the β-globin gene. This point mutation (rs334 T) leads to the production of sickle hemoglobin (HbS), due to the substitution of valine for glutamic acid at the sixth position of the β-globin chain. The hydrophobic residue of valine associates with other hydrophobic residues, which causes HbS molecules to aggregate, forming fibrous precipitates when hemoglobin is deoxygenated. This phenomenon is called “HbS polymerization,” and is responsible for the characteristic shape change, termed “sickling,” of RBCs. Sickle RBC are rigid, and therefore do not easily flow through the microcirculation, causing frequent vaso-occlusive episodes in affected patients. In addition, when RBCs lose their deformability, they become more fragile and prone to hemolysis, which is the root cause of chronic hemolytic anemia in SCA (Connes et al., 2014).

Although the loss of RBC deformability is a fundamental characteristic of SCA, patients exhibit varying degrees of RBC rigidity, which can differentially affect SCA disease severity and complications (Renoux et al., 2016). Newborns usually have almost normal RBC deformability because they still have a high percentage of fetal hemoglobin (HbF) (Renoux et al., 2016). However, when patients become older HbF is replaced by HbS, and, as a result, RBC deformability decreases (Renoux et al., 2016). Hydroxyurea (HU) therapy can affect RBC deformability because HU stimulates HbF synthesis, thereby improving RBC deformability, although the deformability values still remain lower than in the healthy population (Lemonne et al., 2015). In individuals who are not under HU therapy, the presence or absence of alpha-thalassemia can also modulate RBC deformability (Renoux et al., 2017), as the inheritance of alpha-thalassemia results in decreased production of HbS (Rees et al., 2010) and thus lower HbS polymerization.

Rigid, sickle deformed RBC can cause pre-capillary obstruction and contribute to vaso-occlusion (Rees et al., 2010). Furthermore, RBC deformability further decreases during acute vaso-occlusive events. However, patients (adults and children over 5 years of age) who have the highest RBC deformability at steady-state, and who are not under HU therapy, actually have a higher risk of developing frequent painful vaso-occlusive crises, as well as osteonecrosis (Lamarre et al., 2012; Lemonne et al., 2013; Renoux et al., 2017). This paradox seems to be due to the fact that in SCA, RBC with the highest deformability are also the most adherent, which would trigger vaso-occlusion (Ballas et al., 1988). On the other hand, individuals who have improved RBC deformability as a result of HU therapy do not have an elevated risk of vaso-occlusive crisis because HU inhibits RBC, platelets and WBC adhesion to the vascular wall via several mechanisms (Bartolucci et al., 2010; Laurance et al., 2011; Chaar et al., 2014; Pallis et al., 2014; Verger et al., 2014). RBC aggregation is usually lower in SCA patients because of low hematocrit and the inability of irreversible sickle cell to form aggregates. However, the formed RBCs aggregates are reported to be much more robust than in healthy population which could further alter blood flow and tissue perfusion at the pre and post capillary levels (Tripette et al., 2009; Connes et al., 2014).

Sickle cell anemia patients with the highest degree of chronic hemolysis (i.e., those with the most severe anemia) are usually characterized by a severe and chronic reduction of RBC deformability as well as the presence of robust RBC aggregates, and seem to be more prone to develop complications such as leg ulcers, priapism and glomerulopathy (Bartolucci et al., 2012; Connes et al., 2013a; Lamarre et al., 2014). In contrast, patients with the highest RBC deformability tend to have less severe anemia and increased blood viscosity, which could increase the risk of developing frequent vaso-occlusive like complications (Nebor et al., 2011; Lamarre et al., 2012; Charlot et al., 2016). Surprisingly, a recent study did not show a further rise of blood viscosity during acute painful vaso-occlusive crisis (Lapoumeroulie et al., 2019). Instead, the authors found a decrease of RBC deformability, as a result of massive RBC sickling (Ballas and Smith, 1992), and a rise in RBC aggregation and RBC aggregates robustness, which would probably impair blood flow into the microcirculation (Lapoumeroulie et al., 2019).

### Vascular Function Cannot Compensate for the Hemorheological Alterations in SCA

As previously discussed, recent works have demonstrated that increased blood viscosity does not systematically cause a rise in vascular resistance in healthy individuals (Vazquez et al., 2010; Salazar Vazquez et al., 2011). Instead, increasing blood viscosity can facilitate vasodilation and decrease vascular resistance through shear stress-dependent mechanisms, which increase NO production. However, when endothelial/vascular dysfunction is present, like in SCA (Kato et al., 2007; Ataga et al., 2016; Charlot et al., 2016), vasodilation is impaired. Therefore, a rise in blood viscosity cannot be fully compensated for, and could increase the risk of frequent vaso-occlusive crises (Lemonne et al., 2012; Charlot et al., 2016).

Chronic hemolysis is highly implicated in the development of vascular dysfunction in SCA. Once hemoglobin is released into the plasma it autoxidizes, forming free heme, iron, and reactive oxygen species, which cause eNOS uncoupling, and decrease NO bioavailability (Kato et al., 2007). These alterations limit the relaxation of vascular smooth muscle, and contribute to the onset of vaso-occlusive crises (Hebbel et al., 2004). In addition, hemolysis releases the arginase contained in erythrocytes into the plasma. The free arginase hydrolyzes arginine, which is the NO precursor, to ornithine and urea, thereby exacerbating the decrease of NO bioavailability (Kato et al., 2007).

Xanthine oxidase (XO) also contributes to vascular dysfunction in SCA. Aslan et al. (2001) demonstrated that episodes of intrahepatic hypoxia-reoxygenation, which can occur in SCA, induce the release of plasma XO. The released XO can impair vascular function by binding to the vessel luminal cells. This creates an oxidative milieu, which results in NO scavenging via an oxygen free radical-dependent mechanism. Furthermore, Mockesch et al. (2017) recently showed that loss of microvascular function in children with SCA was significantly associated with both nitrotyrosine and markers of systemic oxidative stress. These findings confirm the important roles oxidative stress and NO scavenging play in the development of vascular dysfunction in SCA.

Circulating extra-vesicles, such as microparticles (Camus et al., 2012, 2015) and exosomes (Khalyfa et al., 2016), are also thought to play a role in the development of vascular dysfunction in SCA. Indeed, Camus et al. (2012) demonstrated that infusion of *in vitro*-generated RBC microparticles caused kidney vaso-occlusion in sickle cell mice. Additionally, Khalyfa et al. (2016) demonstrated that exosomes isolated from SCA patients with frequent vaso-occlusive crises decreased endothelial permeability and promoted P-selectin expression on cultured endothelial cells. The exosomes isolated from SCA patients with frequent vaso-occlusive crises also significantly increased the adhesion of monocytes to the vascular wall in mice, compared with exosomes isolated from SCA patients with a less severe phenotype. Overall, these works suggest that therapies focusing both on blood rheology and vascular function could be helpful to decrease the clinical severity of SCA patients.

## Exercise and Blood Rheology in Healthy Individuals and Individuals With SCA

Blood rheology plays an important role in the regulation of tissue perfusion at rest and during exercise. For example, RBCs need to be highly deformable to easily flow through small capillaries and transport oxygen to the tissues (Parthasarathi and Lipowsky, 1999). Any changes in RBC rheological properties during exercise may affect blood viscosity (Baskurt and Meiselman, 2003), which in turn may impact blood flow and exercise performance (Connes et al., 2013b; Waltz et al., 2015). Several investigators have reported significant correlations between blood fluidity and indices of physical fitness in sportsmen, such as time of endurance until exhaustion, aerobic working capacity at 170 W (W170), and maximal oxygen consumption (VO_2__max_) (Ernst et al., 1985; Brun et al., 1986, 1989, 1995).

### The Effect of Acute Cycling Exercise on Blood Viscosity and Its Determinants in Healthy Individuals

**FIGURE 1:**
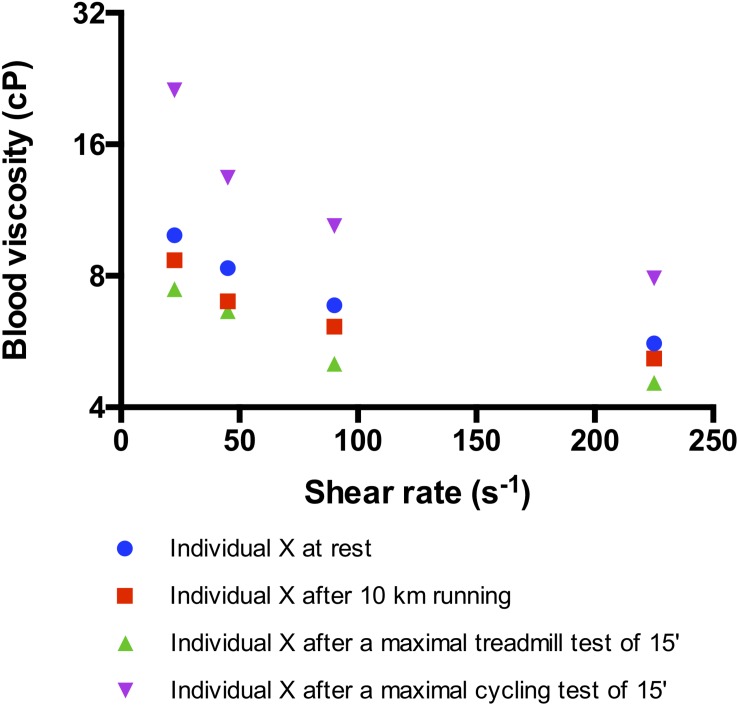
Effects of different kind of exercise on blood viscosity measured at several shear rates in the same trained subject (maximal oxygen consumption, VO_2_max = 64 ml/kg/min). The maximal treadmill and cycling tests consisted in a progressive and maximal exercise conducted until VO_2_max and performed in laboratory conditions (temperature: 24°C). The 10 km running was performed outdoor (20°C) and at the highest intensity the subject could run. Indeed, the subject ran at 77% of his maximal aerobic velocity determined on the treadmill and reached a heart rate of 92% of his maximal heart rate. The subject did not drink water during each test. For the three tests, blood was sampled immediately at the end of the exercise and blood viscosity was measured with the same cone-plate viscometer within 1 h after sampling.

Most of the studies conducted in the eighties and nineties to investigate the acute effects of exercise on blood rheology focused on cycling efforts performed by moderately trained subjects (Brun et al., 1998). These studies reported that acute cycling exercise caused blood viscosity measured at high shear rate (i.e., 100–200 s^–1^) to increase by more than 15% above pre-exercise values (Brun et al., 1998; Connes et al., 2004a, 2013b; Figure 1). This increase in blood viscosity has been attributed to changes in plasma viscosity, hematocrit, and RBC rheological properties (Brun et al., 1998).

Multiple studies have shown that an acute bout of maximal or submaximal cycling exercise may cause plasma viscosity to increase by 10–12%, compared to resting values (Ernst, 1985; Ernst et al., 1991b; Brun et al., 1994b, 1998; Bouix et al., 1998; Connes et al., 2004b). This increase has been attributed to a rise in plasma protein content, such as fibrinogen, α1-globulins, α2-globulins, β-globulins, and γ-globulins during exercise (Nosadova, 1977; Convertino et al., 1981; Vandewalle et al., 1988; Wood et al., 1991).

Several studies have also shown that an acute cycling exercise can result in a 10–12% (i.e., 3–4 units) rise in hematocrit, when compared to pre-exercise values (Brun et al., 1994b; Bouix et al., 1998; Connes et al., 2004a). The effect of elevated hematocrit on blood viscosity at high shear rates has been well described (Cokelet and Meiselman, 2007). A 1-unit increase in hematocrit can cause a 4% increase in blood viscosity. This rise in hematocrit has been attributed to several mechanisms such as fluid shift between intra- and extra-vascular spaces (Sjogaard et al., 1985; Ploutz-Snyder et al., 1995), dehydration (Nosadova, 1977; Stephenson and Kolka, 1988) the release of sequestered RBCs from the spleen (Isbister, 1997), and water trapping in muscle (Ploutz-Snyder et al., 1995).

Acute cycling exercise can also modulate RBC deformability. Several authors have reported a decrease in RBC deformability during and immediately after exercise (Reinhart et al., 1983; Galea and Davidson, 1985; Gueguen-Duchesne et al., 1987; Brun et al., 1993, 1994a; Oostenbrug et al., 1997; Yalcin et al., 2000, 2003). This decrease could be a consequence of lactic acid and reactive oxygen species production that occurs during exercise (Connes et al., 2013b). The accumulation of lactate ions and the decrease in pH would promote RBC dehydration through the activation of several RBC cationic channels, leading to RBC shrinkage and decreased RBC deformability (Van Beaumont et al., 1981; Lipovac et al., 1985; Smith et al., 1997; Connes et al., 2004c). Moreover, several groups have suggested that oxidative stress could also play a role in the decreased RBC deformability through the oxidation of membrane lipid and protein components (Senturk et al., 2005a, b; Connes et al., 2013b). However, the magnitude of the change in RBC deformability that occurs during exercise seems to depend on the training status and physical fitness of the subjects. For instance, Senturk et al. (2005a) reported that a short, progressive, maximal cycling exercise test promoted oxidative stress in both sedentary and well-trained subjects, but they only observed a decrease in RBC deformability and an increase of RBC fragility in sedentary individuals.

Very few studies have observed RBC aggregation during cycling exercise, and the results of these studies are very heterogeneous (Connes et al., 2013b). Yalcin et al. (2003) found that RBC aggregation decreased after a Wingate exercise test. In contrast, Ernst et al. (1991b) observed a transient increase in RBC aggregation above pre-exercise values after 1 h of pedaling at a heart rate of 150 beats min^–1^. RBC aggregation then returned to baseline during the 2 h following the cycling test. Varlet-Marie et al. (2003) and Connes et al. (2007) found no change in RBC aggregation in response to submaximal/maximal cycling exercise. The discrepancies observed are not very well understood and might be related to the (1) kind of exercise performed (short versus prolonged exercise), (2) time of measurement during exercise (i.e., immediately at the end of exercise or few minutes after), (3) time delay for measurement after sampling, and (4) procedure used for RBC aggregation measurement (adequate oxygenation and adjusted hematocrit before measurement or not).

On the whole, these changes lead to a rise in blood viscosity during acute cycling exercise. Large elevations in blood viscosity have generally been considered to be deleterious for the cardiovascular system (Brun et al., 1998; Connes et al., 2013b). However, Connes et al. (2012) reported a positive correlation between the magnitude of change in blood viscosity and the magnitude of change in NO end-stable products, and a negative correlation between the magnitude of change in blood viscosity and the magnitude of change in vascular resistance. These findings suggest that increasing blood viscosity during exercise could be a way to stimulate endothelium-dependent NO production through shear stress-related mechanisms. In healthy individuals, this would result in compensatory vasodilation, thereby preventing any large increases in vascular resistance.

### Acute Running Exercise, Blood Viscosity and Its Determinants in Healthy Individuals

Surprisingly, in contrast to cycling exercises, running exercises, such as a marathon or a 10-km race, do not cause a rise in blood viscosity (Neuhaus et al., 1992; Tripette et al., 2011; Figure 1). The main reason is that hematocrit and plasma viscosity usually remain very stable during these kinds of efforts, despite dehydration (Galea and Davidson, 1985; Neuhaus et al., 1992; Neuhaus and Gaehtgens, 1994). It has been hypothesized that the lack of change in hematocrit could be explained by repeated RBC foot strike hemolysis during running (Neuhaus et al., 1992; Neuhaus and Gaehtgens, 1994; Tripette et al., 2011; Connes et al., 2013b). However, a recent study in which highly trained subjects performed a progressive and maximal treadmill test did not observe any signs of RBC damage or eryptosis (Nader et al., 2018). However, the picture could be slightly different during ultra-running events. For instance, Robach et al. (2014) reported increased hemolysis and large plasma volume expansion immediately after a 166-km long mountain ultra-endurance marathon with 9500 m of altitude gain/loss. The impact of these changes on blood rheology has not been investigated and further studies are needed to address this question.

Surprisingly, Nader et al. (2018) found a slight but significant increase in RBC deformability in response to a short and maximal exercise test. Indeed, although hematocrit increased, blood viscosity remained unchanged (and tended to decrease), as has been observed in previous studies (Neuhaus and Gaehtgens, 1994; Tripette et al., 2011). The findings suggest that the slight increase in RBC deformability could have compensated for the rise in hematocrit observed during short running events, resulting in a lack of change in blood viscosity. The impact of ultra-run events on RBC deformability is unknown. Several other groups found a slight increase in RBC deformability during running (Suhr et al., 2012) and cycling (Connes et al., 2009) exercises. Although the exact reasons for these findings are unknown, it has been suggested that increased NO production during exercise could increase RBC deformability. One of the first works to suggest that NO could affect RBC deformability was the study of Starzyk et al. (1997), which demonstrated that intravenous infusion of L-NAME (an eNOS inhibitor) in rats caused a reduction of RBC deformability. In addition, Bor-Kucukatay et al. (2003) reported that several eNOS inhibitors also decreased RBC deformability, suggesting that basal release of NO actively maintains RBC deformability. While extracellular sources of NO may impact the deformability of RBC, several works suggest that endogenous RBC NO synthesis may also modulate RBC deformability (Kleinbongard et al., 2006). Suhr et al. (2012) demonstrated that an acute running exercise induced shear stress activation of RBC NOS (increased RBC NOS phosphorylation at Ser1177) via the PI3-kinase/Akt kinase pathway, leading to increased NO production by the RBC, which was critical to maintaining RBC deformability during exercise. Grau et al. (2013) further extended these findings by showing that RBC NOS activation by pharmacological treatment (insulin) increased RBC NO content and improved RBC deformability through direct S-nitrosylation of cytoskeleton proteins, most likely the α- and β-spectrins. In contrast, the use of RBC NOS inhibitors [wortmannin or L-N5-(1-Iminoethyl)-ornithin] resulted in a decrease of RBC NOS Ser1177 phosphorylation, NO content, cytoskeleton protein S-nitrosylation, and RBC deformability.

### Impact of Physiological Changes During Acute Exercise on Blood Viscosity

As discussed above, acute cycling exercise would lead to a rise in blood viscosity while running exercise would be characterized by a lack of change in blood viscosity compared to the pre-exercise values. Although methodological aspects could partly explain some of these differences, such as the use of different viscometers (cone-plate viscometer, couette viscometer, capillary viscometer, etc.) or the use of different shear rates (low, moderate, and high shear rate viscosity are affected by different RBC rheological parameters), the picture is probably a little bit more complex. Several physiological changes occur during exercise, which may affect blood viscosity.

To avoid large dehydration during exercise, sportsmen usually drink water or carbohydrate-rich drinks. However, most of the studies performed in the field of blood rheology have been done in laboratory conditions where water is not allowed during the various exercise tests and water loss cannot be compensated by water intake. Tripette et al. (2010) and Diaw et al. (2014) previously tested the impact of add-libitum hydration and water deprivation on blood viscosity during a prolonged submaximal exercise and a soccer game, respectively. Healthy individuals and subjects carrying sickle cell trait were included. While hydration during exercise was able to decrease blood viscosity below the pre-exercise levels in sickle cell trait carriers, blood viscosity increased similarly in healthy individuals in both the “hydration” and “water deprivation” conditions. The rate of dehydration was around 1.5–2% in these studies, which is not very severe. There is a growing interest from the runners community to participate to very prolonged race (>100 kms), sometimes performed at high altitude. The impact of the environment and higher dehydration rate on blood viscosity are unknown and further studies are needed.

Exercise is accompanied by a rise cardiac output and blood flow leading to an increase of shear rate values in the vascular system. For instance, shear rate in the femoral artery has been reported to increase from 60 s^–1^ at rest to 200–250 s^–1^ during exercise (Gonzales et al., 2009). Blood is a shear-thinning fluid, meaning that its viscosity decreases when shear rate increases. Connes et al. (2013b) shown that 15 min of cycling exercise performed at a submaximal intensity increased blood viscosity measured at 90 s^–1^. However, when the viscosity of the blood sampled at the end of exercise was measured at 225 s^–1^ (which reflects the shear rate reached during exercise), the mean value was almost identical to the viscosity of the blood sampled before exercise and measured at 90 s^–1^. Indeed, the effects of hemoconcentration, increased plasma viscosity, decreased RBC deformability and increased RBC aggregation on blood viscosity during exercise could be counterbalanced by the effects of increasing shear rate (Connes et al., 2013b). However, the slight remaining changes in blood viscosity observed during exercise were still correlated with the magnitude of changes in NO-end products concentration, suggesting that blood viscosity plays a role in promoting NO production during exercise (Connes et al., 2013b).

Core temperature rises during exercise and it is well known that blood viscosity also depends on temperature (Baskurt et al., 2009). A recent elegant study shown that when the changes in temperature occurring during exercise are took into account in the measurements of blood viscosity, there is no difference between the pre- and post-exercise values (Buono et al., 2016). The slight physiological hyperthermia was shown to increase RBC deformability, which compensated the rise in hematocrit and resulted in a lack of change in blood viscosity. The authors estimated that the combined effects of increasing shear rate and hyperthermia during exercise could decrease blood viscosity by 31% below the pre-exercise levels, in spite of the exercise induced hemoconcentration (Buono et al., 2016). These findings clearly show that studies in the field of exercise hemorheology should consider the effects of various physiological factors for better interpretation about the role of blood viscosity in the cardiovascular adaptations and physical fitness.

### Long Term Effects of Exercise on Blood Rheology in Healthy Individuals

Chronic exercise (endurance or resistance exercise) usually decreases blood viscosity (Brun et al., 1998; Romain et al., 2011; Kilic-Toprak et al., 2012). One reason for this change is that plasma volume increases few hours or several days after a single bout of exercise (Fellmann, 1992; Brun et al., 1998), resulting in an “autohemodilution” (Ernst, 1987; Ernst et al., 1991a). Repeated exercise bouts on consecutive days leads to a chronic “autohemodilution” resulting in a low baseline hematocrit – low baseline viscosity pattern (Ernst, 1987; Brun et al., 1998; Kilic-Toprak et al., 2012). The magnitude of plasma volume expansion ranges from 9 to 25%, corresponding to an additional 300 to 700 ml of plasma. It has been shown that the larger the reduction in plasma volume during exercise, the greater the subsequent plasma volume expansion (Fellmann, 1992). The amount of water ingested during and after exercise, as well several fluid-regulating hormones (aldosterone, vasopressin and the atrial natriuretic factor) and the level of plasma proteins, influence the degree of the plasma volume expansion after exercise. Plasma volume expansion is responsible for the decrease in plasma viscosity that contributes to the decrease in blood viscosity (Ernst, 1987).

Exercise training also induces RBC rheological adaptations (Brun et al., 2010; Kilic-Toprak et al., 2012). Ernst (1987) reported increased RBC deformability in athletes compared to sedentary subjects, a finding later confirmed by Smith et al. (1999). A 3-month longitudinal study of initially untrained healthy volunteers who performed regular training also showed a decrease of blood viscosity and a rise of RBC deformability (Ernst, 1987). Few studies have been conducted to determine why RBC deformability improves in healthy individuals after chronic exercise. However, Smith et al. (1999) and Tomschi et al. (2018) found a higher proportion of young deformable RBCs in athletes than in untrained subjects. The hemorheological benefits induced by regular exercise are suspected to contribute to the improvements in cardiovascular health that are induced by training programs in patients with cardiovascular disorders (Sandor et al., 2014).

### Acute and Chronic Effects of Exercise in SCA

The metabolic changes occurring during exercise may promote HbS polymerization, RBC sickling, oxidative stress and inflammation. For this reason, physicians have generally been reluctant to promote physical activity for individuals with SCA (Connes, 2010; Waltz and Connes, 2014; Chirico et al., 2016; Martin et al., 2018). However, since regular physical activity has been shown to provide health benefits in various chronic diseases, several groups have begun to study the effects of acute and chronic exercise in individuals and mice with SCA.

Sickle cell anemia patients have decreased aerobic physical fitness compared to the general population (Connes et al., 2011). This is probably due to a combination of several factors, including chronic anemia, reduced muscle mass and strength, abnormal cardiac function, gas exchange abnormalities, mechanical ventilation limitations and peripheral vascular impairment (Callahan et al., 2002; Dougherty et al., 2011; Liem et al., 2015; Badawy et al., 2018; Merlet et al., 2019). A recent study reported negative associations between the oxygen uptake efficiency slope (an index of aerobic physical fitness) and hematocrit, RBC deformability, and RBC aggregate strength in adults with SCA (Charlot et al., 2015). These biological parameters were also associated with the ability of recover from a short submaximal exercise (Charlot et al., 2015). In another study, Waltz et al. (2013) showed that high levels of anemia, low fetal hemoglobin levels, and low RBC deformability were independent predictors of a low 6-min walking test performance.

Blood rheological abnormalities play a key role in the pathophysiology of SCA. For this reason, several works have investigated the effects of acute exercise on various biological parameters to determine what kind of exercise could be considered completely safe for individuals with SCA. In a study performed in Ivory Coast, 17 patients with SCA performed a 20 min moderate exercise (45 Watts) with blood sampling before and at the end of exercise (Balayssac-Siransy et al., 2011; Faes et al., 2014). Despite an increase in the percentages of dense RBC at the end of the effort, blood viscosity and soluble forms of P- and E-selectin remained unchanged compared to the pre-exercise level (Balayssac-Siransy et al., 2011; Faes et al., 2014). Slight increases of the plasma soluble forms of VCAM-1 and ICAM-1 were noted at the end of the exercise in SCA patients, suggesting slight endothelial activation (Faes et al., 2014). On the other hand, another study by Liem et al. (2015) reported higher concentrations of soluble VCAM-1 in the plasma of SCA patients at rest compared to a control group, but progressive and maximal exercise tests did not induce any further rise of VCAM-1. An additional study by Waltz et al. (2012) evaluated blood rheological parameters in subjects with SCA following a short (10–12 min) progressive submaximal cycling exercise, conducted until the first ventilatory threshold was reached. The exercise caused no changes in hematocrit, white blood cell count, blood viscosity, RBC deformability, or RBC aggregation. Furthermore, the strength of RBC aggregates decreased 2 and 3 days after the exercise. This delayed effect of exercise on RBC aggregate strength could be beneficial from a clinical point of view since this parameter is associated with risk of acute chest syndrome (Lamarre et al., 2012), and is increased during vaso-occlusive crises (Lapoumeroulie et al., 2019). Grau et al. (2019) recently confirmed that a short progressive and submaximal cycling exercise had no deleterious effect on RBC deformability and showed that this kind of effort does not exacerbate hemolysis. Finally, Barbeau et al. (2001) previously reported that the repetition of 30 min of moderate exercise for three consecutive days increased plasma NO concentrations in subjects with SCA. This could be viewed as a positive effect since NO bioavailability is reduced in SCA (Kato et al., 2007). It is important to note that none of these studies reported any clinical complications immediately or several days after the exercise (Barbeau et al., 2001; Balayssac-Siransy et al., 2011; Waltz et al., 2012; Faes et al., 2014; Grau et al., 2019). Overall, the results of these studies suggest that mild-to-moderate intensity exercise is probably safe in SCA, but longer or high-intensity exercise should be avoided, or recommended only on a case-by-case basis (Martin et al., 2018). Still, some patients may experience moderate to severe hemoglobin desaturation, even during low-intensity or submaximal exercises, like the 6-min walking test (Waltz et al., 2013). Hemoglobin desaturation can promote RBC sickling in conditions of prolonged hypoxemic stress. Therefore, SCA patients should always be screened for hemoglobin desaturation during exercise tests to identify whether the person is at risk of experiencing exercise-induced hypoxemia during submaximal efforts.

The accumulating evidence showing that acute exercise could be safe for SCA patients prompted several groups to test the effects of regular exercise in mice and humans with SCA. Studies conducted in sickle SAD mice showed that 8 weeks of voluntary wheel running decreased blood viscosity (Faes et al., 2015), limited systemic oxidative stress (Charrin et al., 2015), and decreased pulmonary endothelial activation in response to an hypoxic-reoxygenation stimulus (Aufradet et al., 2014). An additional study conducted by Charrin et al. (2018) evaluated the effects of 8 weeks of aerobic training (1 h/day, 5 days/week) in a more severe mice model of SCA (Townes mice). The chronic exercise decreased several markers of systemic inflammation, including white blood cell count, plasma Th1/Th2 cytokine ratio, and Interleukin-1β level, and reduced the occurrence of splenomegaly. Furthermore, two other studies have also shown that endurance training improved muscle function in Townes SCA mice (Chatel et al., 2018; Gouraud et al., 2019).

Presently, only a few studies have been conducted to evaluate chronic exercise in people with SCA. Omwanghe et al. (2017) recently reported that 90% of children with SCA participate in physical education and 48% participate in sports. These findings illustrate that children with SCA do moderate to vigorous intensity physical activity for short durations. The same research group also prescribed three home exercise sessions per week, for 12 weeks, to 13 children with SCA. Results showed that 77% of subjects completed 89% of the prescribed sessions without any exercise-related adverse events. These results indicate that regular moderate exercise is safe and feasible in children with SCA. In an additional study by Gellen et al. (2018), adults with SCA performed 45 min of exercise three times a week for 8 weeks. No adverse events were reported in the study period, confirming that regular physical activity can be safe for people with SCA. Moreover, the SCA subjects’ power outputs measured at 4 mmol/L blood lactate significantly increased after the 8 weeks training period, indicating improved physical fitness. Currently, no human studies have been conducted to determine whether chronic exercise can modulate the biological parameters (i.e., blood rheology, hematology, inflammation, oxidative stress) that cause the various acute complications experienced by people with SCA. However, the current evidence that exists suggests that moderate-intensity endurance-exercise training could potentially be used as a beneficial therapeutic strategy for patients with SCA (Gellen et al., 2018).

## Conclusion

In conclusion, whole blood viscosity is a physiological parameter that should be considered when studying vascular resistance in healthy populations or in patients affected by various diseases. Plasma viscosity, hematocrit, RBC deformability, and RBC aggregation are all factors that modulate blood viscosity. Alterations in any of these factors can modify blood flow resistance in the vasculature and alter tissue perfusion. In healthy individuals, the vascular system can adapt to elevations in blood viscosity because increased shear stress results in endothelium-dependent NO production. However, in individuals with vascular dysfunction, the vessels are not able to effectively vasodilate. Therefore, elevated blood viscosity can increase vascular resistance.

**FIGURE 2:**
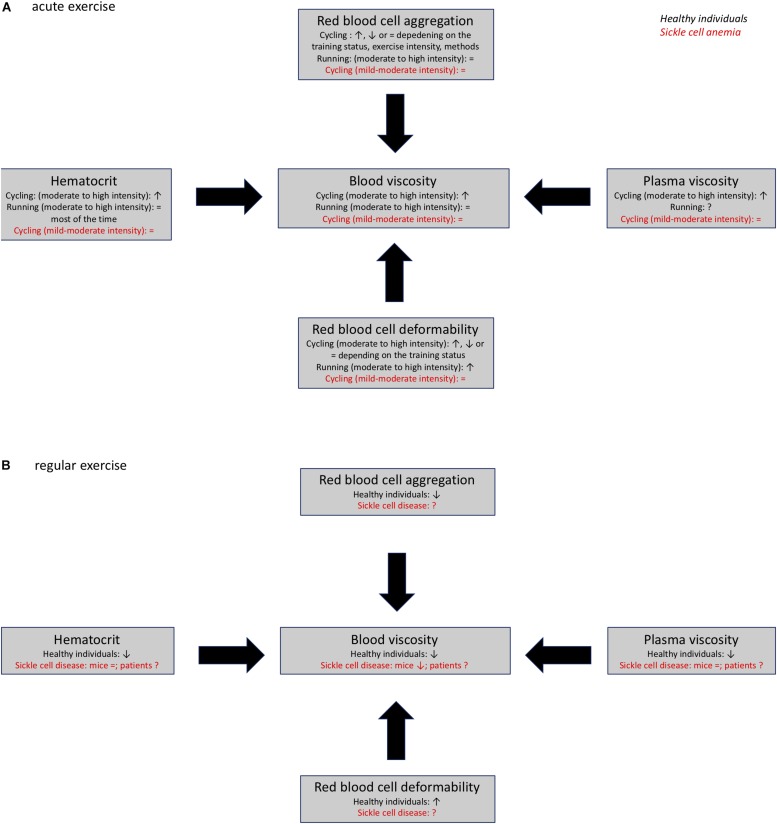
Acute **(A)** and chronic **(B)** effects of exercise on blood rheology (blood viscosity, plasma viscosity, hematocrit, red blood cell deformability, and aggregation) in healthy individuals and patients with sickle cell anemia., ↑, ↓; =, no change; ?, unknown.

Tissue perfusion plays a key role during exercise and exercise can modulate blood viscosity and RBC rheology. The effects of acute exercise in healthy individuals are dependent on exercise modality, intensity, duration and physical fitness of the subjects. Most of the studies have found that acute cycling exercise increases blood viscosity by decreasing RBC deformability and increasing hematocrit and plasma viscosity (Figure 2). In contrast, acute running exercise is suggested to not cause any change in blood viscosity because of unaltered hematocrit (Figure 2). Furthermore, chronic exercise has been shown to decrease blood viscosity by increasing RBC deformability and decreasing hematocrit because of chronic auto-hemodilution (Figure 2).

Sickle cell anemia is a hereditary disease that causes pathological changes in blood rheology that ultimately contribute to the development of vascular dysfunction and disease complications. Several studies have shown that light to moderate intensity acute exercise is well-tolerated in individuals with SCA (Figure 2). Additionally, chronic exercise has been shown to cause positive hemorheological adaptations that could play a role in the positive benefits that result from training programs in patients with various cardiovascular disorders (Figure 2). Therefore, additional studies should be carried out to determine whether chronic exercise could improve blood rheological profiles in individuals with SCA, and decrease the severity of disease complications.

## Author Contributions

EN, SS, and PC wrote the first version of the manuscript. All authors read and approved the final version of the manuscript.

## Conflict of Interest

The authors declare that the research was conducted in the absence of any commercial or financial relationships that could be construed as a potential conflict of interest.

## References

[B1] Aslan M. Ryan T. M. Adler B. Townes T. M. Parks D. A. Thompson J. A. (2001). Oxygen radical inhibition of nitric oxide-dependent vascular function in sickle cell disease. *Proc. Natl. Acad. Sci. U.S.A.* 98 15215–15220. 10.1073/pnas.221292098 11752464 PMC65009

[B2] Ataga K. I. Derebail V. K. Caughey M. Elsherif L. Shen J. H. Jones S. K. (2016). Albuminuria is associated with endothelial dysfunction and elevated plasma endothelin-1 in sickle cell anemia. *PLoS One* 11:e0162652. 10.1371/journal.pone.0162652 27669006 PMC5036885

[B3] Aufradet E. Douillard A. Charrin E. Romdhani A. De Souza G. Bessaad A. (2014). Physical activity limits pulmonary endothelial activation in sickle cell SAD mice. *Blood* 123 2745–2747. 10.1182/blood-2013-10-534982 24764563

[B4] Badawy S. M. Payne A. B. Rodeghier M. J. Liem R. I. (2018). Exercise capacity and clinical outcomes in adults followed in the cooperative study of sickle cell disease (CSSCD). *Eur. J. Haematol.* 101 532–541. 10.1111/ejh.13140 29999202 PMC6546160

[B5] Balayssac-Siransy E. Connes P. Tuo N. Danho C. Diaw M. Sanogo I. (2011). Mild haemorheological changes induced by a moderate endurance exercise in patients with sickle cell anaemia. *Br. J. Haematol.* 154 398–407. 10.1111/j.1365-2141.2011.08728.x 21569006

[B6] Ballas S. K. Larner J. Smith E. D. Surrey S. Schwartz E. Rappaport E. F. (1988). Rheologic predictors of the severity of the painful sickle cell crisis. *Blood* 72 1216–1223. 3167204

[B7] Ballas S. K. Smith E. D. (1992). Red blood cell changes during the evolution of the sickle cell painful crisis. *Blood* 79 2154–2163. 1562742

[B8] Barbeau P. Woods K. F. Ramsey L. T. Litaker M. S. Pollock D. M. Pollock J. S. (2001). Exercise in sickle cell anemia: effect on inflammatory and vasoactive mediators. *Endothelium* 8 147–155. 11572476 10.3109/10623320109165323

[B9] Bartolucci P. Brugnara C. Teixeira-Pinto A. Pissard S. Moradkhani K. Jouault H. (2012). Erythrocyte density in sickle cell syndromes is associated with specific clinical manifestations and hemolysis. *Blood* 120 3136–3141. 10.1182/blood-2012-04-424184 22919030

[B10] Bartolucci P. Chaar V. Picot J. Bachir D. Habibi A. Fauroux C. (2010). Decreased sickle red blood cell adhesion to laminin by hydroxyurea is associated with inhibition of Lu/BCAM protein phosphorylation. *Blood* 116 2152–2159. 10.1182/blood-2009-12-257444 20566895

[B11] Baskurt O. Meiselman H. J. (2007). “In vivo hemorheology,” in *Handbook of Hemorheology and Hemodynamics*, eds Baskurt O. K. Hardeman M. R. Rampling M. W. Meiselman H. J., (Amsterdam: IOS Press), 322–338.

[B12] Baskurt O. K. Boynard M. Cokelet G. C. Connes P. Cooke B. M. Forconi S. (2009). New guidelines for hemorheological laboratory techniques. *Clin. Hemorheol. Microcirc* 42 75–97. 10.3233/CH-2009-1202 19433882

[B13] Baskurt O. K. Meiselman H. J. (2003). Blood rheology and hemodynamics. *Semin. Thromb. Hemost.* 29 435–450. 10.1055/s-2003-44551 14631543

[B14] Baskurt O. K. Meiselman H. J. (2009). Red blood cell “aggregability”. *Clin. Hemorheol. Microcirc* 43 353–354. 10.3233/CH-2009-1255 19996524

[B15] Baskurt O. K. Yalcin O. Meiselman H. J. (2004). Hemorheology and vascular control mechanisms. *Clin. Hemorheol. Microcirc* 30 169–178. 15258340

[B16] Biro K. Sandor B. Kovacs D. Csiszar B. Vekasi J. Totsimon K. (2018). Lower limb ischemia and microrheological alterations in patients with diabetic retinopathy. *Clin. Hemorheol. Microcirc.* 69 23–35. 10.3233/CH-189103 29630532

[B17] Bor-Kucukatay M. Wenby R. B. Meiselman H. J. Baskurt O. K. (2003). Effects of nitric oxide on red blood cell deformability. *Am. J. Physiol. Heart Circ. Physiol.* 284 H1577–H1584. 10.1152/ajpheart.00665.2002 12521942

[B18] Bouix D. Peyreigne C. Raynaud E. Monnier J. F. Micallef J. P. Brun J. F. (1998). Relationships among body composition, hemorheology and exercise performance in rugbymen. *Clin. Hemorheol. Microcirc* 19 245–254. 9874360

[B19] Brun J. F. Criqui C. Orsetti A. (1986). Paramètres hémorhéologiques et exercice physique. *Sports Med. Acta.* 12 56–60.

[B20] Brun J. F. Fons C. Supparo C. Mallard C. Orsetti A. (1993). Could exercise-induced increase in blood viscosity at high shear rate be entirely explained by hematocrit and plasma viscosity changes? *Clin. Hemorheol.* 13 187–199.

[B21] Brun J. F. Khaled S. Raynaud E. Bouix D. Micallef J. P. Orsetti A. (1998). The triphasic effects of exercise on blood rheology: which relevance to physiology and pathophysiology? *Clin. Hemorheol. Microcirc.* 19 89–104. 9849922

[B22] Brun J. F. Micallef J. P. Orsetti A. (1994a). Hemorheologic effects of light prolonged exercise. *Clin. Hemorheol.* 14 807–818.

[B23] Brun J. F. Supparo C. Mallard C. Orsetti A. (1994b). Low values of resting blood viscosity and erythrocyte aggregation are associated with lower increases in blood lactate during submaximal exercise. *Clin. Hemorheol.* 14 105–116.

[B24] Brun J. F. Sekkat M. Lagoueyte C. Fédou C. Orsetti A. (1989). Relashionships between fitness and blood viscosity in untrained normal short children. *Clin. Hemorheol.* 9 953–963.

[B25] Brun J. F. Supparo C. Rama D. Benezis C. Orsetti A. (1995). Maximal oxygen uptake and lactate thresholds during exercise are related to blood viscosity and erythrocyte aggregation in professional football players. *Clin. Hemorheol.* 15 201–212.

[B26] Brun J. F. Varlet-Marie E. Connes P. Aloulou I. (2010). Hemorheological alterations related to training and overtraining. *Biorheology* 47 95–115. 10.3233/BIR-2010-0563 20683154

[B27] Brun J. F. Varlet-Marie E. Richou M. Mercier J. Raynaud De Mauverger E. (2018). Blood rheology as a mirror of endocrine and metabolic homeostasis in health and disease1. *Clin. Hemorheol. Microcirc* 69 239–265. 10.3233/CH-189124 29660919

[B28] Buono M. J. Krippes T. Kolkhorst F. W. Williams A. T. Cabrales P. (2016). Increases in core temperature counterbalance effects of haemoconcentration on blood viscosity during prolonged exercise in the heat. *Exp. Physiol.* 101 332–342. 10.1113/EP085504 26682653 PMC4738148

[B29] Callahan L. A. Woods K. F. Mensah G. A. Ramsey L. T. Barbeau P. Gutin B. (2002). Cardiopulmonary responses to exercise in women with sickle cell anemia. *Am. J. Respir. Crit. Care Med.* 165 1309–1316. 10.1164/rccm.2002036 11991885

[B30] Camus S. M. De Moraes J. A. Bonnin P. Abbyad P. Le Jeune S. Lionnet F. (2015). Circulating cell membrane microparticles transfer heme to endothelial cells and trigger vasoocclusions in sickle cell disease. *Blood* 125 3805–3814. 10.1182/blood-2014-07-589283 25827830 PMC4490297

[B31] Camus S. M. Gausseres B. Bonnin P. Loufrani L. Grimaud L. Charue D. (2012). Erythrocyte microparticles can induce kidney vaso-occlusions in a murine model of sickle cell disease. *Blood* 120 5050–5058. 10.1182/blood-2012-02-413138 22976952

[B32] Chaar V. Laurance S. Lapoumeroulie C. Cochet S. De Grandis M. Colin Y. (2014). Hydroxycarbamide decreases sickle reticulocyte adhesion to resting endothelium by inhibiting endothelial lutheran/basal cell adhesion molecule (Lu/BCAM) through phosphodiesterase 4A activation. *J. Biol. Chem.* 289 11512–11521. 10.1074/jbc.M113.506121 24616094 PMC4036286

[B33] Charlot K. Romana M. Moeckesch B. Jumet S. Waltz X. Divialle-Doumdo L. (2016). Which side of the balance determines the frequency of vaso-occlusive crises in children with sickle cell anemia: Blood viscosity or microvascular dysfunction? *Blood Cells Mol. Dis.* 56 41–45. 10.1016/j.bcmd.2015.10.005 26603723

[B34] Charlot K. Waltz X. Hedreville M. Sinnapah S. Lemonne N. Etienne-Julan M. (2015). Impaired oxygen uptake efficiency slope and off-transient kinetics of pulmonary oxygen uptake in sickle cell anemia are associated with hemorheological abnormalities. *Clin. Hemorheol. Microcirc.* 60 413–421. 10.3233/CH-141891 25261432

[B35] Charrin E. Aufradet E. Douillard A. Romdhani A. Souza G. D. Bessaad A. (2015). Oxidative stress is decreased in physically active sickle cell SAD mice. *Br. J. Haematol.* 168 747–756. 10.1111/bjh.13207 25382268

[B36] Charrin E. Dube J. J. Connes P. Pialoux V. Ghosh S. Faes C. (2018). Moderate exercise training decreases inflammation in transgenic sickle cell mice. *Blood Cells Mol. Dis.* 69 45–52. 10.1016/j.bcmd.2017.06.002 28624257

[B37] Chatel B. Messonnier L. A. Barge Q. Vilmen C. Noirez P. Bernard M. (2018). Endurance training reduces exercise-induced acidosis and improves muscle function in a mouse model of sickle cell disease. *Mol. Genet. Metab.* 123 400–410. 10.1016/j.ymgme.2017.11.010 29307759

[B38] Chien S. King R. G. Skalak R. Usami S. Copley A. L. (1975). Viscoelastic properties of human blood and red cell suspensions. *Biorheology* 12 341–346. 1212514 10.3233/bir-1975-12603

[B39] Chien S. Usami S. Dellenback R. J. Gregersen M. I. (1970). Shear-dependent deformation of erythrocytes in rheology of human blood. *Am. J. Physiol.* 219 136–142. 10.1152/ajplegacy.1970.219.1.136 5424839

[B40] Chirico E. N. Faes C. Connes P. Canet-Soulas E. Martin C. Pialoux V. (2016). Role of exercise-induced oxidative stress in sickle cell trait and disease. *Sports Med.* 46 629–639. 10.1007/s40279-015-0447-z 26666745

[B41] Clark M. R. Mohandas N. Shohet S. B. (1983). Osmotic gradient ektacytometry: comprehensive characterization of red cell volume and surface maintenance. *Blood* 61 899–910. 6831052

[B42] Cokelet G. R. Goldsmith H. L. (1991). Decreased hydrodynamic resistance in the two-phase flow of blood through small vertical tubes at low flow rates. *Circ. Res.* 68 1–17. 10.1161/01.res.68.1.1 1984854

[B43] Cokelet G. R. Meiselman H. J. (2007). “Macro- and micro-rheological properties of blood,” in *Handbook of Hemorheology and Hemodynamics*, eds Baskurt O. K. Hardeman M. R. Rampling M. W. Meiselman H. J., (Amsterdam: IOS Press), 45–71.

[B44] Connes P. (2010). Hemorheology and exercise: effects of warm environments and potential consequences for sickle cell trait carriers. *Scand. J. Med. Sci. Sports* 20(Suppl. 3), 48–52. 10.1111/j.1600-0838.2010.01208.x 21029190

[B45] Connes P. Alexy T. Detterich J. Romana M. Hardy-Dessources M. D. Ballas S. K. (2016). The role of blood rheology in sickle cell disease. *Blood Rev.* 30 111–118. 10.1016/j.blre.2015.08.005 26341565 PMC6447059

[B46] Connes P. Bouix D. Durand F. Kippelen P. Mercier J. Prefaut C. (2004a). Is hemoglobin desaturation related to blood viscosity in athletes during exercise? *Int. J. Sports Med.* 25 569–574. 15531998 10.1055/s-2004-821118

[B47] Connes P. Bouix D. Py G. Caillaud C. Kippelen P. Brun J. F. (2004b). Does exercise-induced hypoxemia modify lactate influx into erythrocytes and hemorheological parameters in athletes? *J. Appl. Physiol.* 97 1053–1058. 15121747 10.1152/japplphysiol.00993.2003

[B48] Connes P. Bouix D. Py G. Préfaut C. Mercier J. Brun J. F. (2004c). Opposite effects of in vitro lactate on erythrocyte deformability in athletes and untrained subjects. *Clin. Hemorheol. Microcirc* 31 311–318. 15567902

[B49] Connes P. Caillaud C. Py G. Mercier J. Hue O. Brun J. F. (2007). Maximal exercise and lactate do not change red blood cell aggregation in well trained athletes. *Clin. Hemorheol. Microcirc* 36 319–326. 17502702

[B50] Connes P. Hue O. Tripette J. Hardy-Dessources M. D. (2008). Blood rheology abnormalities and vascular cell adhesion mechanisms in sickle cell trait carriers during exercise. *Clin. Hemorheol. Microcirc* 39 179–184. 18503123

[B51] Connes P. Lamarre Y. Hardy-Dessources M. D. Lemonne N. Waltz X. Mougenel D. (2013a). Decreased hematocrit-to-viscosity ratio and increased lactate dehydrogenase level in patients with sickle cell anemia and recurrent leg ulcers. *PLoS One* 8:e79680. 10.1371/journal.pone.0079680 24223994 PMC3817120

[B52] Connes P. Simmonds M. J. Brun J. F. Baskurt O. K. (2013b). Exercise hemorheology: classical data, recent findings and unresolved issues. *Clin. Hemorheol. Microcirc* 53 187–199. 10.3233/CH-2012-1643 23042105

[B53] Connes P. Lamarre Y. Waltz X. Ballas S. K. Lemonne N. Etienne-Julan M. (2014). Haemolysis and abnormal haemorheology in sickle cell anaemia. *Br. J. Haematol.* 165 564–572. 10.1111/bjh.12786 24611951

[B54] Connes P. Machado R. Hue O. Reid H. (2011). Exercise limitation, exercise testing and exercise recommendations in sickle cell anemia. *Clin. Hemorheol. Microcirc.* 49 151–163. 10.3233/CH-2011-1465 22214686

[B55] Connes P. Pichon A. Hardy-Dessources M. D. Waltz X. Lamarre Y. Simmonds M. J. (2012). Blood viscosity and hemodynamics during exercise. *Clin. Hemorheol. Microcirc.* 51 101–109. 10.3233/CH-2011-1515 22240371

[B56] Connes P. Tripette J. Mukisi-Mukaza M. Baskurt O. K. Toth K. Meiselman H. J. (2009). Relationships between hemodynamic, hemorheological and metabolic responses during exercise. *Biorheology* 46 133–143. 10.3233/BIR-2009-0529 19458416

[B57] Convertino V. A. Keil L. C. Bernauer E. M. Greenleaf J. E. (1981). Plasma volume, osmolality, vasopressin, and renin activity during graded exercise in man. *J. Appl. Physiol. Respir. Environ. Exerc. Physiol.* 50 123–128. 10.1152/jappl.1981.50.1.123 7009522

[B58] Diaw M. Samb A. Diop S. Sall N. D. Ba A. Cisse F. (2014). Effects of hydration and water deprivation on blood viscosity during a soccer game in sickle cell trait carriers. *Br. J. Sports Med.* 48 326–331. 10.1136/bjsports-2012-091038 22685122

[B59] Dougherty K. A. Schall J. I. Rovner A. J. Stallings V. A. Zemel B. S. (2011). Attenuated maximal muscle strength and peak power in children with sickle cell disease. *J. Pediatr. Hematol. Oncol.* 33 93–97. 10.1097/MPH.0b013e318200ef49 21228717 PMC3078038

[B60] Dupire J. Socol M. Viallat A. (2012). Full dynamics of a red blood cell in shear flow. *Proc. Natl. Acad. Sci. U.S.A.* 109 20808–20813. 10.1073/pnas.1210236109 23213229 PMC3529085

[B61] Ernst E. (1985). Changes in blood rheology produced by exercise. *J. Am. Med. Assoc.* 253 2962–2963. 3999274

[B62] Ernst E. (1987). Influence of regular physical activity on blood rheology. *Eur. Heart J.* 8(Suppl. G), 59–62. 3443127 10.1093/eurheartj/8.suppl_g.59

[B63] Ernst E. Danburger L. Saradeth T. (1991a). Changes in plasma volume after prolonged endurance exercise. *Med. Sci. Sports Exerc.* 23:884. 1921684

[B64] Ernst E. Daburger L. Saradeth T. (1991b). The kinetics of blood rheology during and after prolonged standardized exercise. *Clin Hemorheol.* 11 429–439.

[B65] Ernst E. Matrai A. Aschenbrenner E. Will V. Schmidlechner C. (1985). Relationship between fitness and blood fluidity. *Clin. Hemorheol.* 5 507–510.

[B66] Faes C. Balayssac-Siransy E. Connes P. Hivert L. Danho C. Bogui P. (2014). Moderate endurance exercise in patients with sickle cell anaemia: effects on oxidative stress and endothelial activation. *Br. J. Haematol.* 164 124–130. 10.1111/bjh.12594 24903630

[B67] Faes C. Charrin E. Connes P. Pialoux V. Martin C. (2015). Chronic physical activity limits blood rheology alterations in transgenic SAD mice. *Am. J. Hematol.* 90 E32–E33. 10.1002/ajh.23896 25382738

[B68] Fellmann N. (1992). Hormonal and plasma volume alterations following endurance exercise. A brief review. *Sports Med.* 13 37–49. 10.2165/00007256-199213010-00004 1553454

[B69] Fischer T. M. Stohr-Lissen M. Schmid-Schonbein H. (1978). The red cell as a fluid droplet: tank tread-like motion of the human erythrocyte membrane in shear flow. *Science* 202 894–896. 10.1126/science.715448 715448

[B70] Galea G. Davidson R. J. (1985). Hemorrheology of marathon running. *Int. J. Sports Med.* 6 136–138. 10.1055/s-2008-1025826 4030187

[B71] Gellen B. Messonnier L. A. Galacteros F. Audureau E. Merlet A. N. Rupp T. (2018). Moderate-intensity endurance-exercise training in patients with sickle-cell disease without severe chronic complications (EXDRE): an open-label randomised controlled trial. *Lancet Haematol.* 5 e554–e562. 10.1016/S2352-3026(18)30163-7 30389037

[B72] Goldsmith H. L. Frank J. M. Maclntosh C. (1972). Flow behaviour of erythrocytes. Rotation and deformation in dilute suspensions. *Proc. R. Soc. B.* 182 351–384.

[B73] Gonzales J. U. Parker B. A. Ridout S. J. Smithmyer S. L. Proctor D. N. (2009). Femoral shear rate response to knee extensor exercise: an age and sex comparison. *Biorheology* 46 145–154. 10.3233/BIR-2009-0535 19458417

[B74] Gouraud E. Charrin E. Dube J. J. Ofori-Acquah S. F. Martin C. Skinner S. (2019). Effects of individualized treadmill endurance training on oxidative stress in skeletal muscles of transgenic sickle mice. *Oxid. Med. Cell Longev.* 2019:3765643. 10.1155/2019/3765643 31428225 PMC6681588

[B75] Grau M. Jerke M. Nader E. Schenk A. Renoux C. Collins B. (2019). Effect of acute exercise on RBC deformability and RBC nitric oxide synthase signalling pathway in young sickle cell anaemia patients. *Sci. Rep.* 9:11813. 10.1038/s41598-019-48364-1 31413300 PMC6694163

[B76] Grau M. Pauly S. Ali J. Walpurgis K. Thevis M. Bloch W. (2013). RBC-NOS-dependent S-nitrosylation of cytoskeletal proteins improves RBC deformability. *PLoS One* 8:e56759. 10.1371/journal.pone.0056759 23424675 PMC3570529

[B77] Gueguen-Duchesne M. Durand F. Beillot J. Dezier J. Rochcongar P. Legoff M. (1987). Could maximal exercise be a hemorheological risk factor? *Clin. Hemorheol.* 7 418.

[B78] Hakim T. S. (1988). Erythrocyte deformability and segmental pulmonary vascular resistance: osmolarity and heat treatment. *J. Appl. Physiol.* 65 1634–1641. 10.1152/jappl.1988.65.4.1634 3182528

[B79] Hebbel R. P. Osarogiagbon R. Kaul D. (2004). The endothelial biology of sickle cell disease: inflammation and a chronic vasculopathy. *Microcirculation* 11 129–151. 15280088

[B80] Intaglietta M. (2009). Increased blood viscosity: disease, adaptation or treatment? *Clin. Hemorheol. Microcirc.* 42 305–306. 10.3233/CH-2009-1236 19628897

[B81] Isbister J. P. (1997). Physiology and pathophysiology of blood volume regulation. *Transfus. Sci.* 18 409–423. 10175155 10.1016/S0955-3886(97)00040-4

[B82] Kato G. J. Gladwin M. T. Steinberg M. H. (2007). Deconstructing sickle cell disease: reappraisal of the role of hemolysis in the development of clinical subphenotypes. *Blood Rev.* 21 37–47. 10.1016/j.blre.2006.07.001 17084951 PMC2048670

[B83] Kesmarky G. Kenyeres P. Rabai M. Toth K. (2008). Plasma viscosity: a forgotten variable. *Clin. Hemorheol. Microcirc.* 39 243–246. 18503132

[B84] Khalyfa A. Khalyfa A. A. Akbarpour M. Connes P. Romana M. Lapping-Carr G. (2016). Extracellular microvesicle microRNAs in children with sickle cell anaemia with divergent clinical phenotypes. *Br. J. Haematol.* 174 786–798. 10.1111/bjh.14104 27161653

[B85] Kilic-Toprak E. Ardic F. Erken G. Unver-Kocak F. Kucukatay V. Bor-Kucukatay M. (2012). Hemorheological responses to progressive resistance exercise training in healthy young males. *Med. Sci. Monit.* 18 CR351–CR360. 10.12659/msm.882878 22648250 PMC3560717

[B86] Kleinbongard P. Schulz R. Rassaf T. Lauer T. Dejam A. Jax T. (2006). Red blood cells express a functional endothelial nitric oxide synthase. *Blood* 107 2943–2951. 10.1182/blood-2005-10-3992 16368881

[B87] Ko E. Youn J. M. Park H. S. Song M. Koh K. H. Lim C. H. (2018). Early red blood cell abnormalities as a clinical variable in sepsis diagnosis. *Clin. Hemorheol. Microcirc.* 70 355–363. 10.3233/CH-180430 30320561

[B88] Lamarre Y. Romana M. Lemonne N. Hardy-Dessources M. D. Tarer V. Mougenel D. (2014). Alpha thalassemia protects sickle cell anemia patients from macro-albuminuria through its effects on red blood cell rheological properties. *Clin. Hemorheol. Microcirc.* 57 63–72. 10.3233/CH-131772 24004554

[B89] Lamarre Y. Romana M. Waltz X. Lalanne-Mistrih M. L. Tressieres B. Divialle-Doumdo L. (2012). Hemorheological risk factors of acute chest syndrome and painful vaso-occlusive crisis in children with sickle cell disease. *Haematologica* 97 1641–1647. 10.3324/haematol.2012.066670 22689686 PMC3487435

[B90] Lanotte L. Mauer J. Mendez S. Fedosov D. A. Fromental J. M. Claveria V. (2016). Red cells’ dynamic morphologies govern blood shear thinning under microcirculatory flow conditions. *Proc. Natl. Acad. Sci. U.S.A.* 113 13289–13294. 10.1073/pnas.1608074113 27834220 PMC5127344

[B91] Lapoumeroulie C. Connes P. El Hoss S. Hierso R. Charlot K. Lemonne N. (2019). New insights into red cell rheology and adhesion in patients with sickle cell anaemia during vaso-occlusive crises. *Br. J. Haematol.* 185 991–994. 10.1111/bjh.15686 30467840

[B92] Laurance S. Lansiaux P. Pellay F. X. Hauchecorne M. Benecke A. Elion J. (2011). Differential modulation of adhesion molecule expression by hydroxycarbamide in human endothelial cells from the micro- and macrocirculation: potential implications in sickle cell disease vasoocclusive events. *Haematologica* 96 534–542. 10.3324/haematol.2010.026740 21228039 PMC3069230

[B93] Lemonne N. Charlot K. Waltz X. Ballas S. K. Lamarre Y. Lee K. (2015). Hydroxyurea treatment does not increase blood viscosity and improves red blood cell rheology in sickle cell anemia. *Haematologica* 100 e383–e386. 10.3324/haematol.2015.130435 26137960 PMC4591770

[B94] Lemonne N. Connes P. Romana M. Vent-Schmidt J. Bourhis V. Lamarre Y. (2012). Increased blood viscosity and red blood cell aggregation in a patient with sickle cell anemia and smoldering myeloma. *Am. J. Hematol.* 87:E129. 10.1002/ajh.23312 22930533

[B95] Lemonne N. Lamarre Y. Romana M. Mukisi-Mukaza M. Hardy-Dessources M. D. Tarer V. (2013). Does increased red blood cell deformability raise the risk for osteonecrosis in sickle cell anemia? *Blood* 121 3054–3056. 10.1182/blood-2013-01-480277 23580637 PMC3988032

[B96] Liem R. I. Reddy M. Pelligra S. A. Savant A. P. Fernhall B. Rodeghier M. (2015). Reduced fitness and abnormal cardiopulmonary responses to maximal exercise testing in children and young adults with sickle cell anemia. *Physiol. Rep.* 3:e12338. 10.14814/phy2.12338 25847915 PMC4425953

[B97] Lipovac V. Gavella M. Turck Z. Skrabalo Z. (1985). Influence of lactate on the insulin action on red blood cell filterability. *Clin. Hemorheol.* 5 421–428.

[B98] Martin C. Pialoux V. Faes C. Charrin E. Skinner S. Connes P. (2018). Does physical activity increase or decrease the risk of sickle cell disease complications? *Br. J. Sports Med.* 52 214–218. 10.1136/bjsports-2015-095317 26701924

[B99] Martini J. Carpentier B. Negrete A. C. Frangos J. A. Intaglietta M. (2005). Paradoxical hypotension following increased hematocrit and blood viscosity. *Am. J. Physiol. Heart Circ. Physiol.* 289 H2136–H2143. 16006543 10.1152/ajpheart.00490.2005

[B100] Meiselman H. J. Neu B. Rampling M. W. Baskurt O. K. (2007). RBC aggregation: laboratory data and models. *Indian J. Exp. Biol.* 45 9–17. 17249322

[B101] Merlet A. N. Chatel B. Hourde C. Ravelojaona M. Bendahan D. Feasson L. (2019). How sickle cell disease impairs skeletal muscle function: implications in daily life. *Med. Sci. Sports Exerc.* 51 4–11. 10.1249/MSS.0000000000001757 30095751

[B102] Mockesch B. Connes P. Charlot K. Skinner S. Hardy-Dessources M. D. Romana M. (2017). Association between oxidative stress and vascular reactivity in children with sickle cell anaemia and sickle haemoglobin C disease. *Br. J. Haematol.* 178 468–475. 10.1111/bjh.14693 28466542

[B103] Nader E. Connes P. Lamarre Y. Renoux C. Joly P. Hardy-Dessources M. D. (2017). Plasmapheresis may improve clinical condition in sickle cell disease through its effects on red blood cell rheology. *Am. J. Hematol.* 92 E629–E630. 10.1002/ajh.24870 28741718

[B104] Nader E. Guillot N. Lavorel L. Hancco I. Fort R. Stauffer E. (2018). Eryptosis and hemorheological responses to maximal exercise in athletes: comparison between running and cycling. *Scand. J. Med. Sci. Sports* 28 1532–1540. 10.1111/sms.13059 29356101

[B105] Nebor D. Bowers A. Hardy-Dessources M. D. Knight-Madden J. Romana M. Reid H. (2011). Frequency of pain crises in sickle cell anemia and its relationship with the sympatho-vagal balance, blood viscosity and inflammation. *Haematologica* 96 1589–1594. 10.3324/haematol.2011.047365 21750084 PMC3208675

[B106] Neuhaus D. Behn C. Gaehtgens P. (1992). Haemorheology and exercise: intrinsic flow properties of blood in marathon running. *Int. J. Sports Med.* 13 506–511. 10.1055/s-2007-1021307 1459744

[B107] Neuhaus D. Gaehtgens P. (1994). Haemorrheology and long term exercise. *Sports Med.* 18 10–21. 10.2165/00007256-199418010-00003 7939036

[B108] Nosadova J. (1977). The changes in hematocrit, hemoglobin, plasma volume and proteins during and after different types of exercise. *Eur. J. Appl. Physiol.* 36 223–230. 10.1007/BF00421753 870321

[B109] Omwanghe O. A. Muntz D. S. Kwon S. Montgomery S. Kemiki O. Hsu L. L. (2017). Self-Reported physical activity and exercise patterns in children with sickle cell disease. *Pediatr. Exerc. Sci.* 29 388–395. 10.1123/pes.2016-0276 28530510

[B110] Oostenbrug G. S. Mensink R. P. Hardeman M. R. De Vries T. Brouns F. Hornstra G. (1997). Exercise performance, red blood cell deformability, and lipid peroxidation: effects of fish oil and vitamin E. *J. Appl. Physiol.* 83 746–752. 9292459 10.1152/jappl.1997.83.3.746

[B111] Pallis F. R. Conran N. Fertrin K. Y. Olalla Saad S. T. Costa F. F. Franco-Penteado C. F. (2014). Hydroxycarbamide reduces eosinophil adhesion and degranulation in sickle cell anaemia patients. *Br. J. Haematol.* 164 286–295. 10.1111/bjh.12628 24383847

[B112] Parthasarathi K. Lipowsky H. H. (1999). Capillary recruitment in response to tissue hypoxia and its dependence on red blood cell deformability. *Am. J. Physiol.* 277 H2145–H2157. 10.1152/ajpheart.1999.277.6.H2145 10600832

[B113] Piecuch J. Mertas A. Nowowiejska-Wiewiora A. Zurawel R. Gregorczyn S. Czuba Z. (2019). The relationship between the rheological behavior of RBCs and angiogenesis in the morbidly obese. *Clin. Hemorheol. Microcirc.* 71 95–102. 10.3233/CH-180420 30530969

[B114] Piel F. B. Patil A. P. Howes R. E. Nyangiri O. A. Gething P. W. Williams T. N. (2010). Global distribution of the sickle cell gene and geographical confirmation of the malaria hypothesis. *Nat. Commun.* 1:104. 10.1038/ncomms1104 21045822 PMC3060623

[B115] Ploutz-Snyder L. L. Convertino V. A. Dudley G. A. (1995). Resistance exercise-induced fluid shifts: change in active muscle size and plasma volume. *Am. J. Physiol.* 269 R536–R543. 7573553 10.1152/ajpregu.1995.269.3.R536

[B116] Poiseuille J. L. M. (1835). Recherches sur les causes du mouvement du sang dans les vaisseaux capillaires. *C R Acad. Sci. Paris* 1 554–560.

[B117] Pop G. A. Duncker D. J. Gardien M. Vranckx P. Versluis S. Hasan D. (2002). The clinical significance of whole blood viscosity in (cardio)vascular medicine. *Neth. Heart J.* 10 512–516. 25696056 PMC2499821

[B118] Raj J. U. Kaapa P. Hillyard R. Anderson J. (1991). Pulmonary vascular pressure profile in adult ferrets: measurements in vivo and in isolated lungs. *Acta Physiol. Scand.* 142 41–48. 10.1111/j.1748-1716.1991.tb09126.x 1877364

[B119] Rampling M. W. Meiselman H. J. Neu B. Baskurt O. K. (2004). Influence of cell-specific factors on red blood cell aggregation. *Biorheology* 41 91–112. 15090679

[B120] Rees D. C. Williams T. N. Gladwin M. T. (2010). Sickle-cell disease. *Lancet* 376 2018–2031. 10.1016/S0140-6736(10)61029-X 21131035

[B121] Reinhart W. H. Staubli M. Straub P. W. (1983). Impaired red cell filterability with elimination of old red blood cells during a 100-km race. *J. Appl. Physiol. Respir. Environ. Exerc. Physiol.* 54 827–830. 10.1152/jappl.1983.54.3.827 6841229

[B122] Renoux C. Connes P. Nader E. Skinner S. Faes C. Petras M. (2017). Alpha-thalassaemia promotes frequent vaso-occlusive crises in children with sickle cell anaemia through haemorheological changes. *Pediatr. Blood Cancer* 64:e26455. 10.1002/pbc.26455 28097791

[B123] Renoux C. Faivre M. Bessaa A. Da Costa L. Joly P. Gauthier A. (2019). Impact of surface-area-to-volume ratio, internal viscosity and membrane viscoelasticity on red blood cell deformability measured in isotonic condition. *Sci. Rep.* 9:6771. 10.1038/s41598-019-43200-y 31043643 PMC6494803

[B124] Renoux C. Romana M. Joly P. Ferdinand S. Faes C. Lemonne N. (2016). Effect of age on blood rheology in sickle cell anaemia and sickle cell haemoglobin C disease: a cross-sectional study. *PLoS One* 11:e0158182. 10.1371/journal.pone.0158182 27355589 PMC4927160

[B125] Robach P. Boisson R. C. Vincent L. Lundby C. Moutereau S. Gergele L. (2014). Hemolysis induced by an extreme mountain ultra-marathon is not associated with a decrease in total red blood cell volume. *Scand. J. Med. Sci. Sports.* 24 18–27. 10.1111/j.1600-0838.2012.01481.x 22672635

[B126] Romain A. J. Brun J. F. Varlet-Marie E. Raynaud De Mauverger E. (2011). Effects of exercise training on blood rheology: a meta-analysis. *Clin. Hemorheol. Microcirc.* 49 199–205. 10.3233/CH-2011-1469 22214690

[B127] Salazar Vazquez B. Y. Cabrales P. Tsai A. G. Intaglietta M. (2011). Nonlinear cardiovascular regulation consequent to changes in blood viscosity. *Clin. Hemorheol. Microcirc.* 49 29–36. 10.3233/CH-2011-1454 22214675

[B128] Sandor B. Nagy A. Toth A. Rabai M. Mezey B. Csatho A. (2014). Effects of moderate aerobic exercise training on hemorheological and laboratory parameters in ischemic heart disease patients. *PLoS One* 9:e110751. 10.1371/journal.pone.0110751 25347067 PMC4210208

[B129] Schmid-Schonbein H. Wells R. E. Goldstone J. (1969). [Model experiments on erythrocyte rheology]. *Pflugers. Arch.* 312 R39–R40. 5390246

[B130] Senturk U. K. Gunduz F. Kuru O. Kocer G. Ozkaya Y. G. Yesilkaya A. (2005a). Exercise-induced oxidative stress leads hemolysis in sedentary but not trained humans. *J. Appl. Physiol.* 99 1434–1441. 15976356 10.1152/japplphysiol.01392.2004

[B131] Senturk U. K. Yalcin O. Gunduz F. Kuru O. Meiselman H. J. Baskurt O. K. (2005b). Effect of antioxidant vitamin treatment on the time course of hematological and hemorheological alterations after an exhausting exercise episode in human subjects. *J. Appl. Physiol.* 98 1272–1279. 15579575 10.1152/japplphysiol.00875.2004

[B132] Sheremet’ev Y. A. Popovicheva A. N. Rogozin M. M. Levin G. Y. (2019). Red blood cell aggregation, disaggregation and aggregate morphology in autologous plasma and serum in diabetic foot disease. *Clin. Hemorheol. Microcirc.* 72 221–227. 10.3233/CH-180405 30909193

[B133] Sjogaard G. Adams R. P. Saltin B. (1985). Water and ion shifts in skeletal muscle of humans with intense dynamic knee extension. *Am. J. Physiol.* 248 R190–R196. 3970234 10.1152/ajpregu.1985.248.2.R190

[B134] Smith J. A. Martin D. T. Telford R. D. Ballas S. K. (1999). Greater erythrocyte deformability in world-class endurance athletes. *Am. J. Physiol.* 276 H2188–H2193. 10.1152/ajpheart.1999.276.6.H2188 10362703

[B135] Smith J. A. Telford R. D. Kolbuch-Braddon M. Weidemann M. J. (1997). Lactate/H+ uptake by red blood cells during exercise alters their physical properties. *Eur. J. Appl. Physiol. Occup. Physiol.* 75 54–61. 9007458 10.1007/s004210050126

[B136] Sriram K. Salazar Vazquez B. Y. Tsai A. G. Cabrales P. Intaglietta M. Tartakovsky D. M. (2012). Autoregulation and mechanotransduction control the arteriolar response to small changes in hematocrit. *Am. J. Physiol. Heart Circ. Physiol.* 303 H1096–H1106. 10.1152/ajpheart.00438.2012 22923620 PMC3517642

[B137] Starzyk D. Korbut R. Gryglewski R. J. (1997). The role of nitric oxide in regulation of deformability of red blood cells in acute phase of endotoxaemia in rats. *J. Physiol. Pharmacol.* 48 731–735. 9444620

[B138] Stephenson L. A. Kolka M. A. (1988). Plasma volume during heat stress and exercise in women. *Eur. J. Appl. Physiol. Occup. Physiol.* 57 373–381. 3396550 10.1007/BF00417979

[B139] Suhr F. Brenig J. Muller R. Behrens H. Bloch W. Grau M. (2012). Moderate exercise promotes human RBC-NOS activity, NO production and deformability through Akt kinase pathway. *PLoS One* 7:e45982. 10.1371/journal.pone.0045982 23049912 PMC3457942

[B140] Tomschi F. Bizjak D. Bloch W. Latsch J. Predel H. G. Grau M. (2018). Deformability of different red blood cell populations and viscosity of differently trained young men in response to intensive and moderate running. *Clin. Hemorheol. Microcirc.* 69 503–514. 10.3233/CH-189202 29710695

[B141] Totsimon K. Biro K. Szabo Z. E. Toth K. Kenyeres P. Marton Z. (2017). The relationship between hemorheological parameters and mortality in critically ill patients with and without sepsis. *Clin. Hemorheol. Microcirc.* 65 119–129. 10.3233/CH-16136 27447421

[B142] Tripette J. Alexy T. Hardy-Dessources M. D. Mougenel D. Beltan E. Chalabi T. (2009). Red blood cell aggregation, aggregate strength and oxygen transport potential of blood are abnormal in both homozygous sickle cell anemia and sickle-hemoglobin C disease. *Haematologica* 94 1060–1065. 10.3324/haematol.2008.005371 19644138 PMC2719028

[B143] Tripette J. Hardy-Dessources M. D. Beltan E. Sanouiller A. Bangou J. Chalabi T. (2011). Endurance running trial in tropical environment: a blood rheological study. *Clin. Hemorheol. Microcirc* 47 261–268. 10.3233/CH-2011-1388 21654055

[B144] Tripette J. Loko G. Samb A. Gogh B. D. Sewade E. Seck D. (2010). Effects of hydration and dehydration on blood rheology in sickle cell trait carriers during exercise. *Am. J. Physiol. Heart Circ. Physiol.* 299 H908–H914. 10.1152/ajpheart.00298.2010 20581085

[B145] Tsai A. G. Acero C. Nance P. R. Cabrales P. Frangos J. A. Buerk D. G. (2005). Elevated plasma viscosity in extreme hemodilution increases perivascular nitric oxide concentration and microvascular perfusion. *Am. J. Physiol. Heart Circ. Physiol.* 288 H1730–H1739. 10.1152/ajpheart.00998.2004 15576432

[B146] Van Beaumont W. Underkofler S. Van Beaumont S. (1981). Erythrocyte volume, plasma volume, and acid-base changes in exercise and heat dehydration. *J. Appl. Physiol. Respir. Environ. Exerc. Physiol.* 50 1255–1262. 10.1152/jappl.1981.50.6.1255 7263386

[B147] Vandewalle H. Lacombe C. Lelievre J. C. Poirot C. (1988). Blood viscosity after a 1-h submaximal exercise with and without drinking. *Int. J. Sports Med.* 9 104–107. 3384514 10.1055/s-2007-1024988

[B148] Varlet-Marie E. Gaudard A. Monnier J. F. Micallef J. P. Mercier J. Bressolle F. (2003). Reduction of red blood cell disaggregability during submaximal exercise: relationship with fibrinogen levels. *Clin. Hemorheol. Microcirc* 28 139–149. 12775896

[B149] Vazquez B. Y. Vazquez M. A. Jaquez M. G. Huemoeller A. H. Intaglietta M. Cabrales P. (2010). Blood pressure directly correlates with blood viscosity in diabetes type 1 children but not in normals. *Clin. Hemorheol. Microcirc* 44 55–61. 10.3233/CH-2010-1252 20134093

[B150] Verger E. Schoevaert D. Carrivain P. Victor J. M. Lapoumeroulie C. Elion J. (2014). Prior exposure of endothelial cells to hydroxycarbamide alters the flow dynamics and adhesion of sickle red blood cells. *Clin. Hemorheol. Microcirc* 57 9–22. 10.3233/CH-131762 24002118

[B151] Waltz X. Connes P. (2014). Pathophysiology and physical activity in patients with sickle cell anemia. *Mov. Sport Sci. Sci. Motricité* 83 41–47. 10.1051/sm/2013105

[B152] Waltz X. Hardy-Dessources M. D. Lemonne N. Mougenel D. Lalanne-Mistrih M. L. Lamarre Y. (2015). Is there a relationship between the hematocrit-to-viscosity ratio and microvascular oxygenation in brain and muscle? *Clin. Hemorheol. Microcirc.* 59 37–43. 10.3233/CH-131742 23719422

[B153] Waltz X. Hedreville M. Sinnapah S. Lamarre Y. Soter V. Lemonne N. (2012). Delayed beneficial effect of acute exercise on red blood cell aggregate strength in patients with sickle cell anemia. *Clin. Hemorheol. Microcirc.* 52 15–26. 10.3233/CH-2012-1540 22414551

[B154] Waltz X. Romana M. Lalanne-Mistrih M. L. Machado R. F. Lamarre Y. Tarer V. (2013). Hematologic and hemorheological determinants of resting and exercise-induced hemoglobin oxygen desaturation in children with sickle cell disease. *Haematologica* 98 1039–1044. 10.3324/haematol.2013.083576 23539539 PMC3696606

[B155] Wood S. C. Doyle M. P. Appenzeller O. (1991). Effects of endurance training and long distance running on blood viscosity. *Med. Sci. Sports Exerc.* 23 1265–1269. 1766342

[B156] Yalcin O. Bor-Kucukatay M. Senturk U. K. Baskurt O. K. (2000). Effects of swimming exercise on red blood cell rheology in trained and untrained rats. *J. Appl. Physiol.* 88 2074–2080. 10846020 10.1152/jappl.2000.88.6.2074

[B157] Yalcin O. Erman A. Muratli S. Bor-Kucukatay M. Baskurt O. K. (2003). Time course of hemorheological alterations after heavy anaerobic exercise in untrained human subjects. *J. Appl. Physiol.* 94 997–1002. 12391137 10.1152/japplphysiol.00368.2002

[B158] Yalcin O. Meiselman H. J. Armstrong J. K. Baskurt O. K. (2005). Effect of enhanced red blood cell aggregation on blood flow resistance in an isolated-perfused guinea pig heart preparation. *Biorheology* 42 511–520. 16369087

